# Supplementary material for: Arrhythmia risk predictions from molecular simulations of cardiac ion channel-drug interactions
Source: Biophys J. 2025 Dec 16;125(16):4177–91. doi: 10.1016/j.bpj.2025.12.015 (PMC13507445; doi:10.1016/j.bpj.2025.12.015)
Supplement: Supplementary file 1 [file mmc1.pdf]

Biophysical Journal, Volume *125*

Supplemental information

Arrhythmia risk predictions from molecular simulations of cardiac ion channel-drug interactions

Kyle C. Rouen, Kush Narang, Yanxiao Han, David Wang, Ensley Jang, Sophia Brunkow, Vladimir Yarov-Yarovoy, Alexander D. MacKerell Jr., and Igor Vorobyov

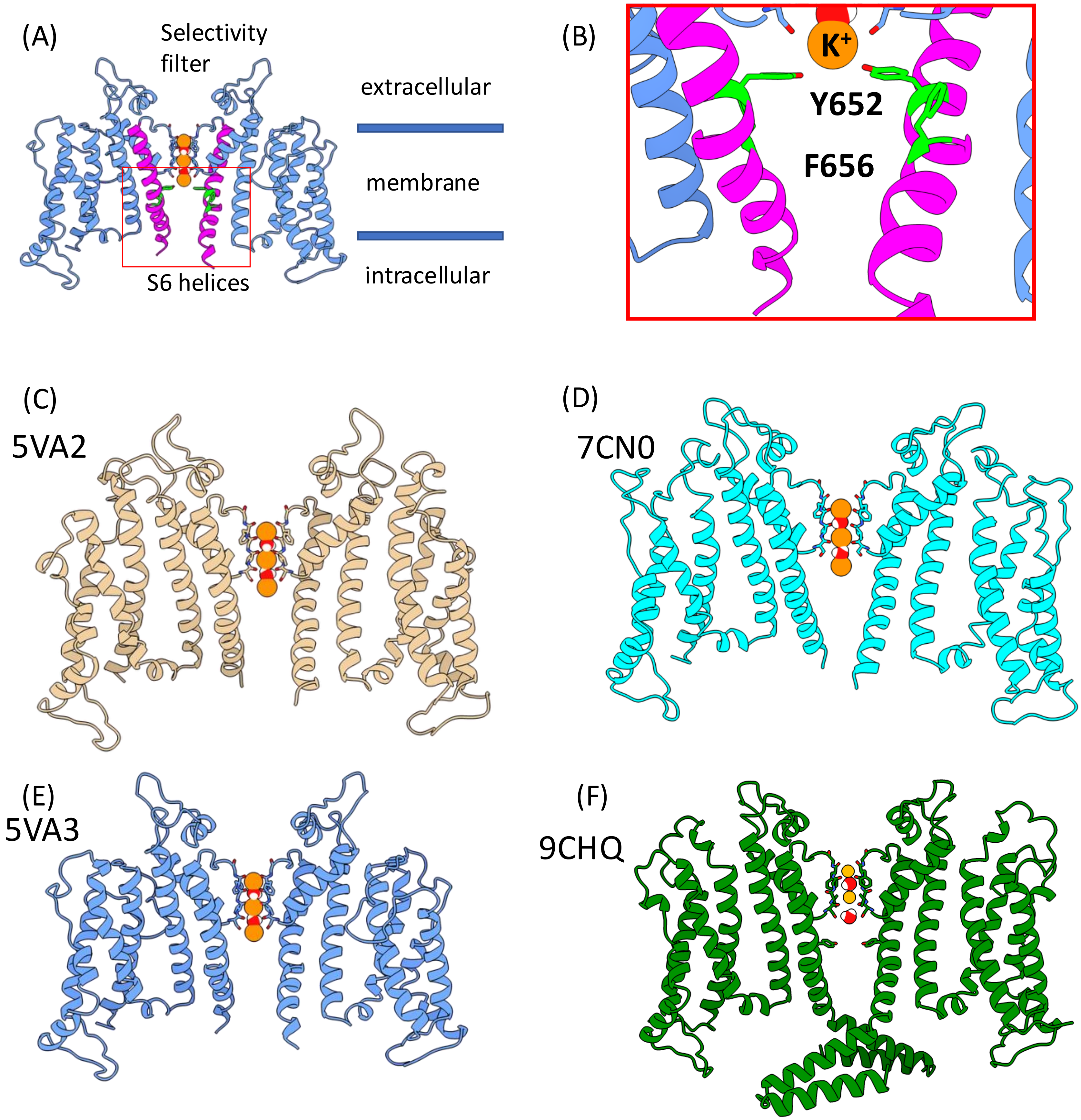

Figure S1: **Structural models of the hERG (K_V11.1) channel.** (A) Structure of the channel with two opposite subunits relative to the lipid membrane position. (B) Notable drug binding residues Y652, F656 relative to the K^+ ion binding site. (C) Structural model of the open state based on PDB: 5VA2 (D) Structural model of the inactivated state based on PDB: 7CN0. (E) Structural model of the non-inactivating mutant based on PDB: 5VA3. (F) Structural model of the low K^+ WT hERG channel based on PDB: 9CHQ.

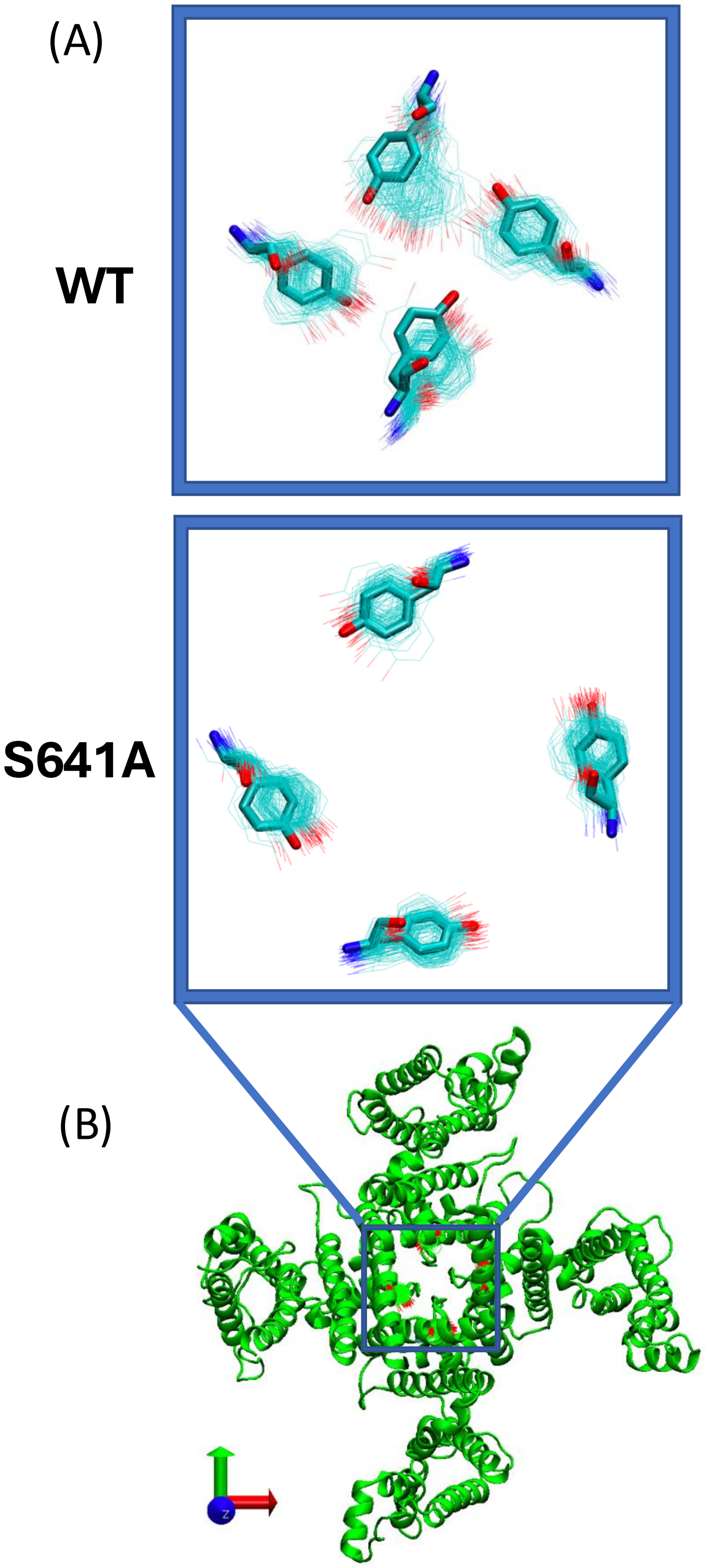

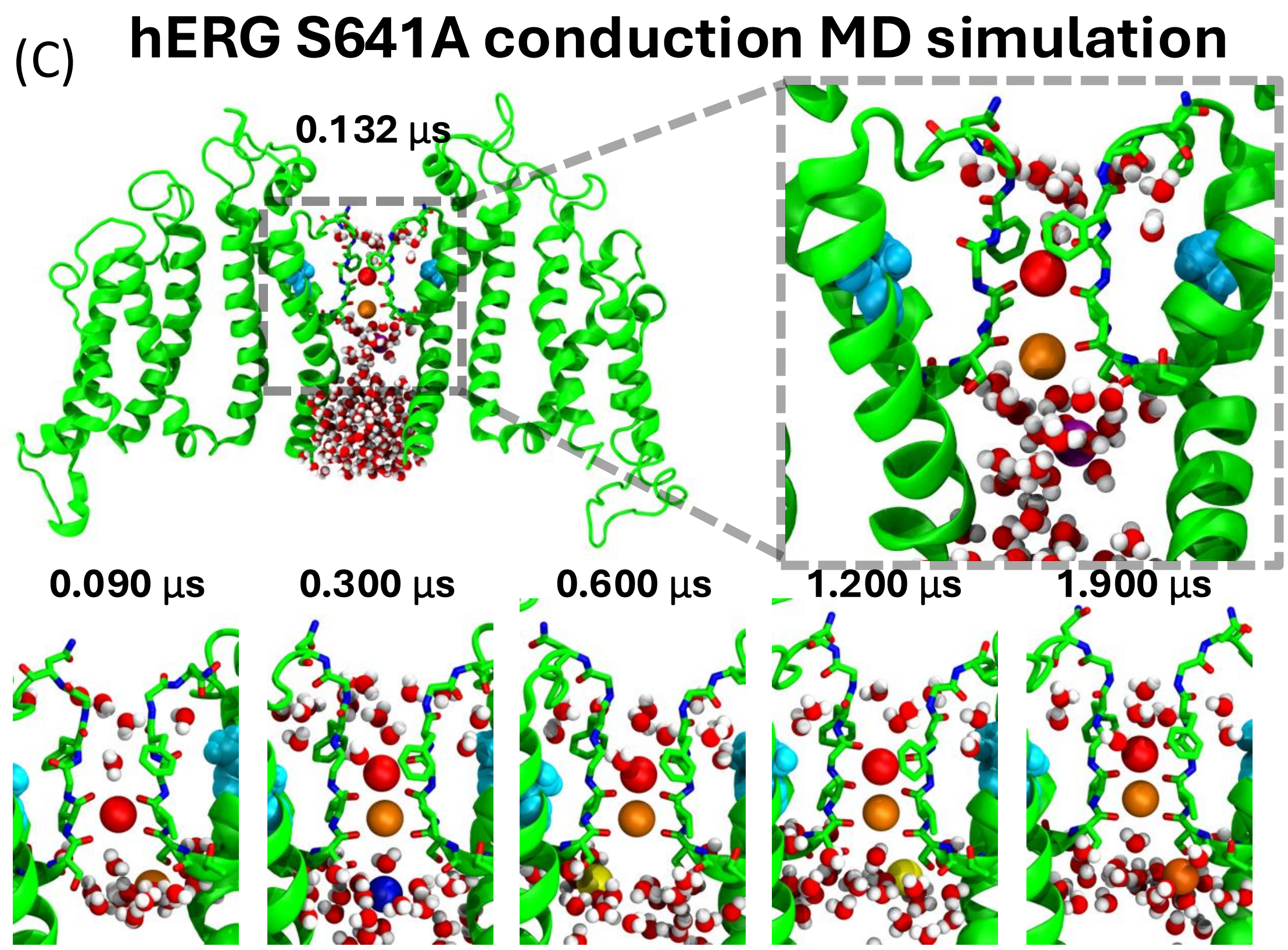

***Figure S2:* Analysis of hERG S641A mutant model**. (A) Orientation of Tyr 652 residues with initial positions shown as thick side chains and every 100th frame from SILCS FragMap-generating simulations shown as thin sticks. (B) hERG channel structural model perspective from intracellular side looking in the positive *z* direction (C) *Top row:* Representative molecular dynamics (MD) simulation frame showing two opposite hERG channel chains (green ribbons) with selectivity filter (SF) S624-G628, S6 helix Y652 and F656 residues shown as sticks with red O and blue N, mutated Ala 641 residue shown as cyan in a space-filling representation; pore ions (colored spheres); and water molecules (red/white). Inset on the right shows close-up view with SF pinched at the top clearly visible. *Bottom row:* Close-up views of the channel SF in the same representation at different MD simulation time points, showing no K^+ ion translocation for this hERG S641A mutant during a 2 μs simulation with applied 750 mV voltage.

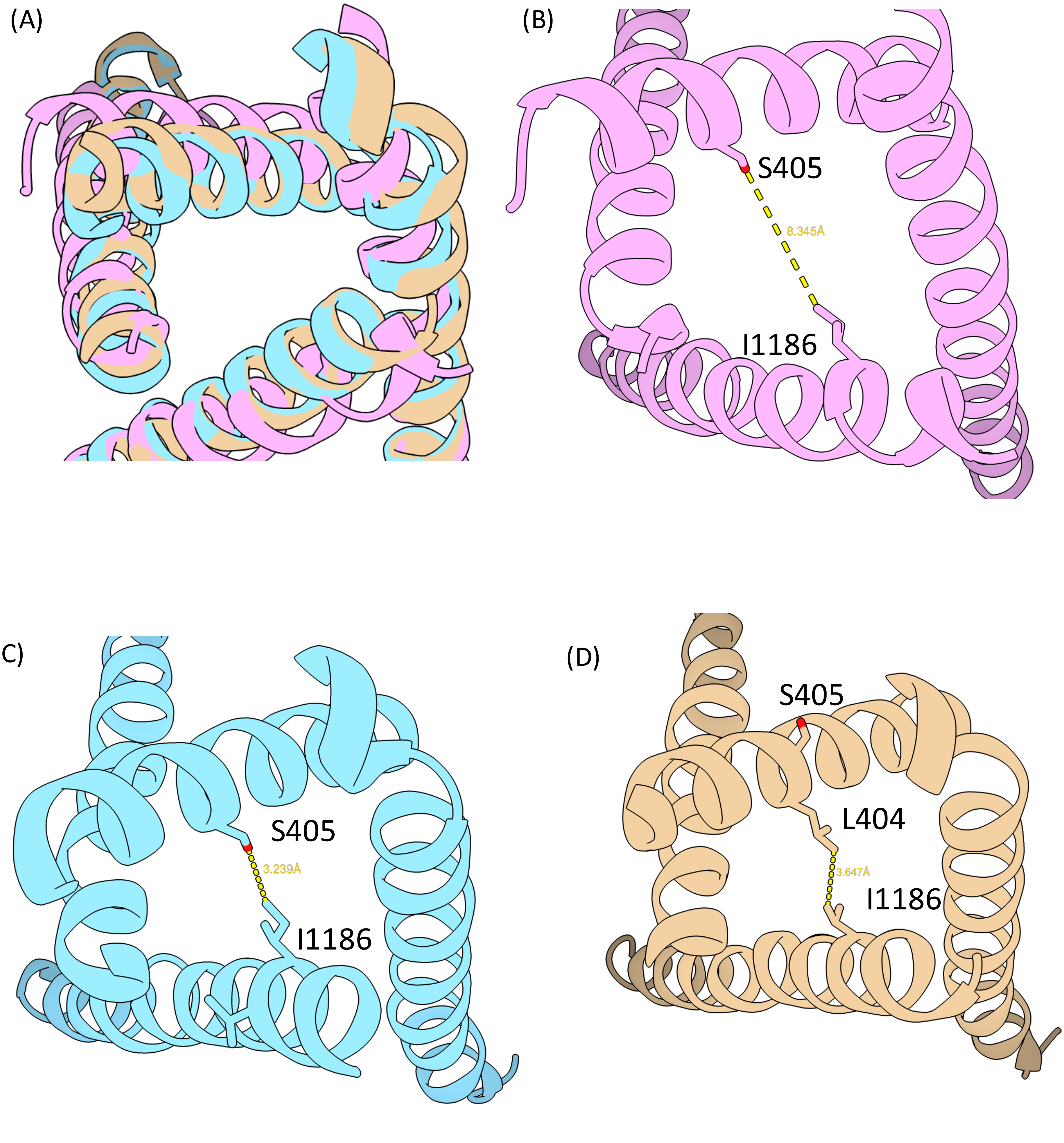

Figure S3: **Ca_V1.2 channel structural models**. (A) Overlay of pore-lining S6 helices for 3 Ca_V1.2 models including (B) Homology model based on PDB: 6AGF (pink), (C) PDB: 8FHS model (blue) and (D) PDB: 8FD7 model (brown).

Model Name	Starting PDB ID	Notes	Preparation
hERG_5VA2	5VA2	Conducting state	Extracellular loops rebuilt with Rosetta
hERG_7CN0	7CN0	Similar state to PDB: 5VA2	Extracellular loops rebuilt with Rosetta
hERG_9CHQ	9CHQ	Cryo-EM performed under low [K^+] to produce more inactivated conformation	Extracellular loops rebuilt with Rosetta
hERG_S631A	5VA3	Non-inactivating mutant, likely open conformation	Extracellular loops rebuilt with Rosetta
hERG_S641A	5VA2	Fast-inactivating mutant	Starting from hERG_5VA2 model, the S641A mutation added with Rosetta, then relaxed with MD simulation
Cav_6AGF	6AGF	Wider intracellular pore	Rosetta comparative modeling used to generate $Ca_V1.2$
Cav_8FHS	8FHS	Inactivated conformation, narrow pore, amiodarone bound in the pore	Auxiliary subunits and ligands removed
Cav_8FD7	8FD7	Gabapentin bound to the auxiliary $\alpha_2\delta$ subunit	Auxiliary subunits and ligands removed
Nav_5XSY	5XSY	Inactivated conformation	Rosetta comparative modeling used to generate $Na_V1.5$
Nav_6LQA	6LQA	Quinidine bound in the channel pore	Glycosylation removed from extracellular residues

***Figure S4:* Cardiac ion channel structural models investigated.** For more details on model preparation, see *Materials and Methods* section in the main text.

(A)

Param Set	Gauss1	Gauss2	Repulsion	Hydrophobic	Hydrogen Bond	Rotation
Default	−0.0356	−0.00516	0.840	-0.0351	-0.587	0.0585
Pham et al.	-0.049811	-0.007218	0.756221	-0.031562	-0.469951	0.025722
hERG optimized	-0.049811	-0.007218	0.756221	-0.04559	-0.469951	0.025722

(B)

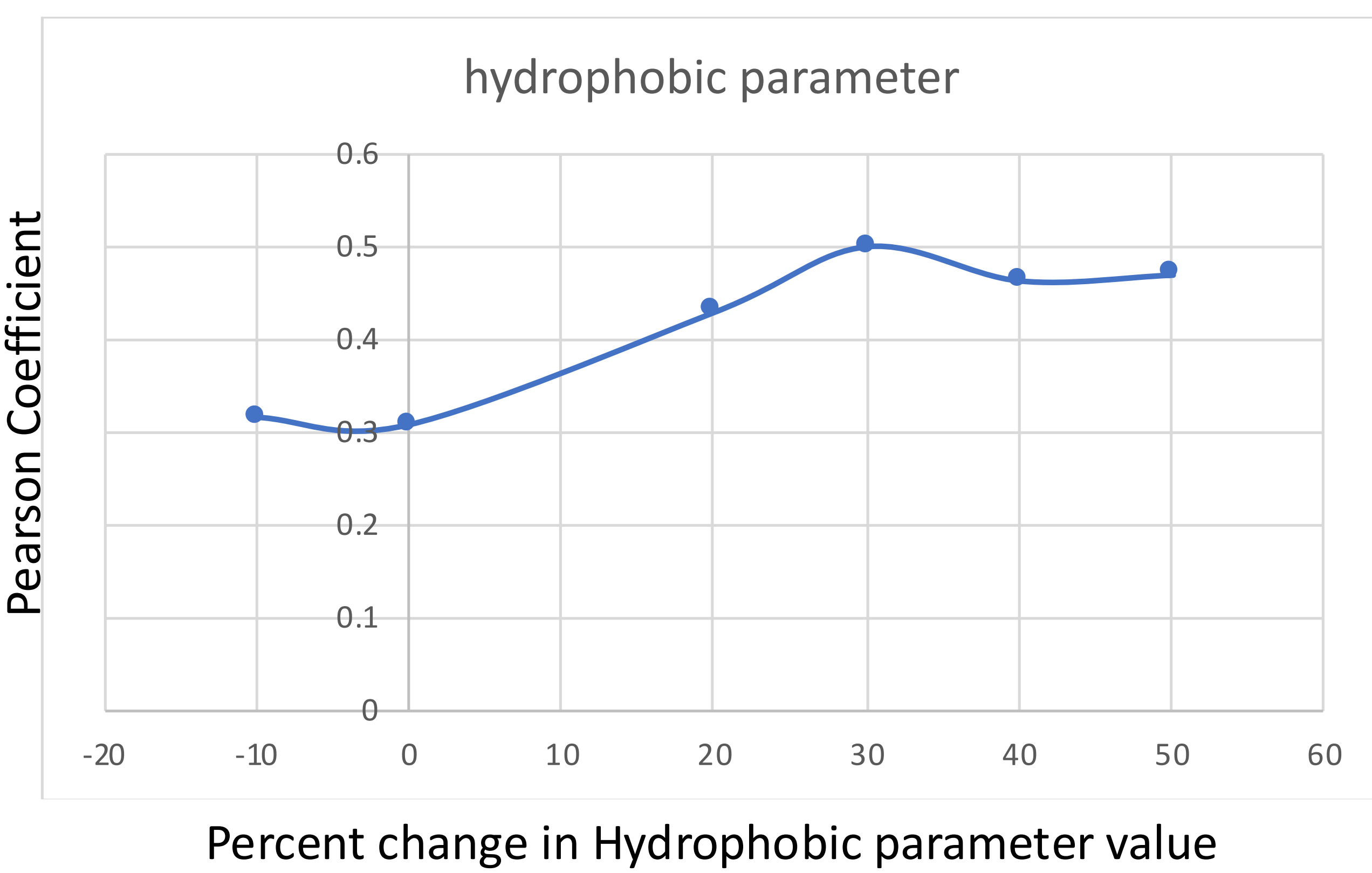

Figure S5: **AutoDock Vina parameter optimization.** (A) Optimized parameter set for the hERG channel with increased hydrophobic weight highlighted in yellow and (B) Correlation of computed and experimental binding free energies for the test drugs as a function of the hydrophobic parameter.

(a)	Coefficient	*p*-value
hERG_5VA2	-0.760	0.005
hERG_9CHQ	-0.166	0.5
intercept	-9.774	0.001

(b)	Coefficient	*p*-value
hERG_5VA2	-0.982	0.006
Cav_6AGF	-0.932	0.004
intercept	-16.502	0.002

(c)	Coefficient	*p*-value
hERG_5VA2	-1.62	0.003
hERG_9CHQ	-0.804	0.03
hERG_S641A	1.78	0.01
intercept	-10.243	0.005

(d)	Coefficient	*p*-value
hERG_5VA2	-3.139	0.007
Nav_6LQA	1.164	0.01
Cav_6AGF	-2.108	0.003
intercept	-29.204	0.004

(e)	Coefficient	*p*-value
hERG_5VA2	-5.124	0.03
hERG_S641A	4.636	0.03
Cav_6AGF	-3.468	0.04
intercept	-37.776	0.05

(f)	Coefficient	*p*-value
hERG_5VA2	-1.868	0.003
hERG_9CHQ	1.281	0.02
Cav_6AGF	-2.228	0.005
intercept	-21.650	0.004

Figure S6: **Terms from logistic regression analysis using leave-one-out cross validation on 53-drug training set.** Model performance is shown in the main-text Figure 3. The *p*-values were computed in R (*glm*, binomial family) using two-sided Wald tests of logistic regression coefficients.

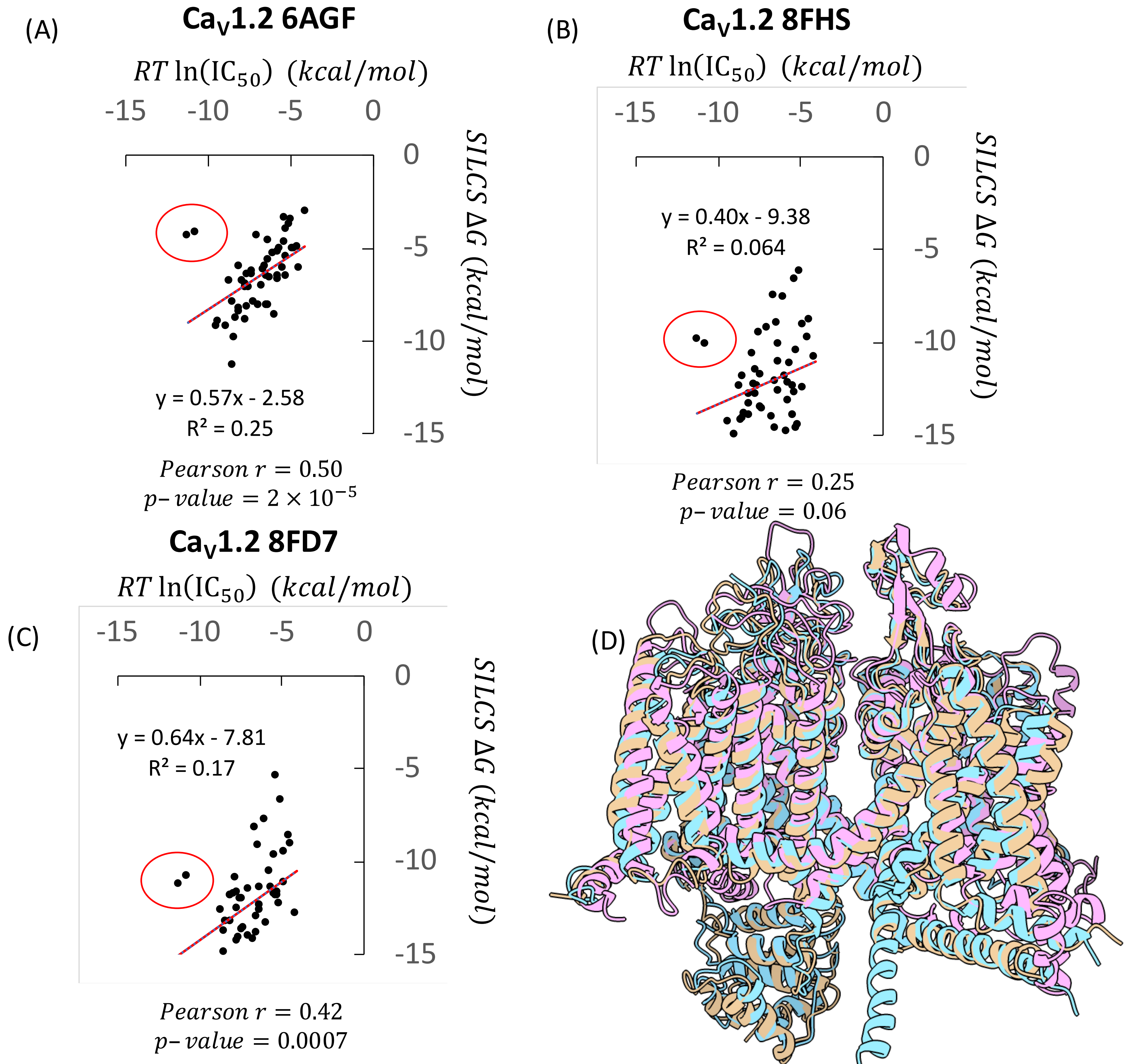

Figure S7: **SILCS docking of 55 hERG-blocking drugs compared to experimental affinities from Kramer et al. (main-text ref. 19).** (A) Experimental affinity vs docking score to the Ca_V1.2 homology model based on PDB: 6AGF. (B) Experimental affinity vs docking score to the Ca_V1.2 PDB: 8FHS model. (C) Experimental affinity vs docking score to the Ca_V1.2 PDB: 8FD7 model. Scores for nifedipine and nitrendipine are circled in each panel. (D) Alignment of PDB: 6AGF (pink), 8FHS (blue), and 8FD7 (pink) Ca_V1.2 structural models. The *p*-values were computed using two-tailed Pearson correlation tests under the null hypothesis of zero linear correlation between docking scores (ΔG) and experimental affinity ($RT \ln IC_{50}$) measurements.

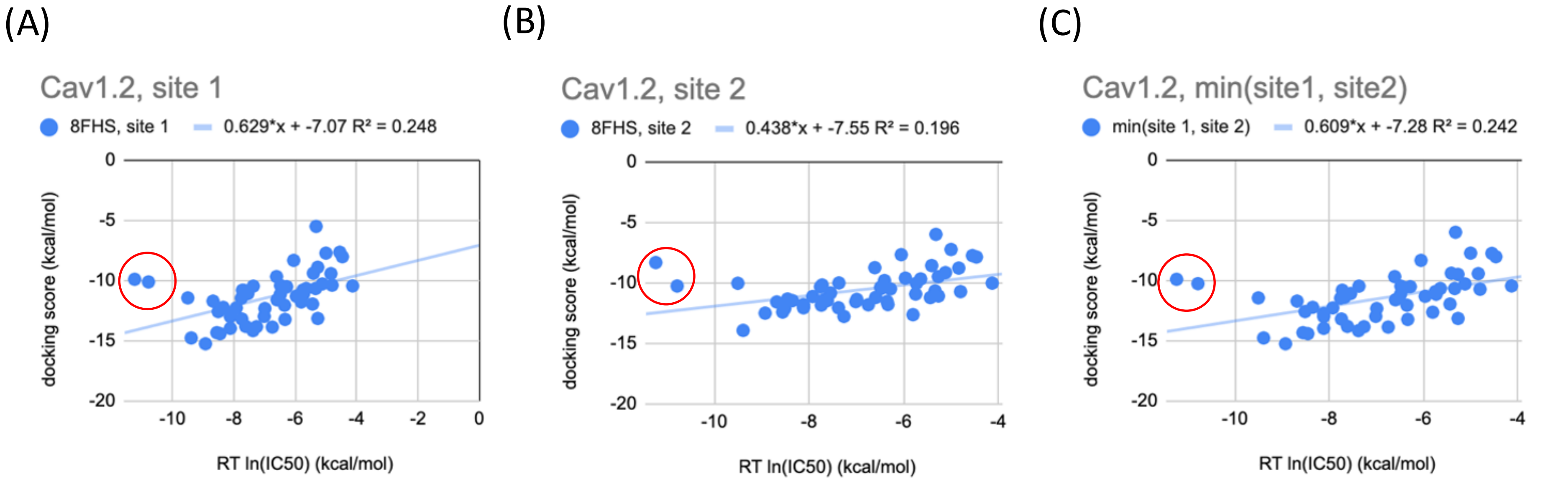

(D)

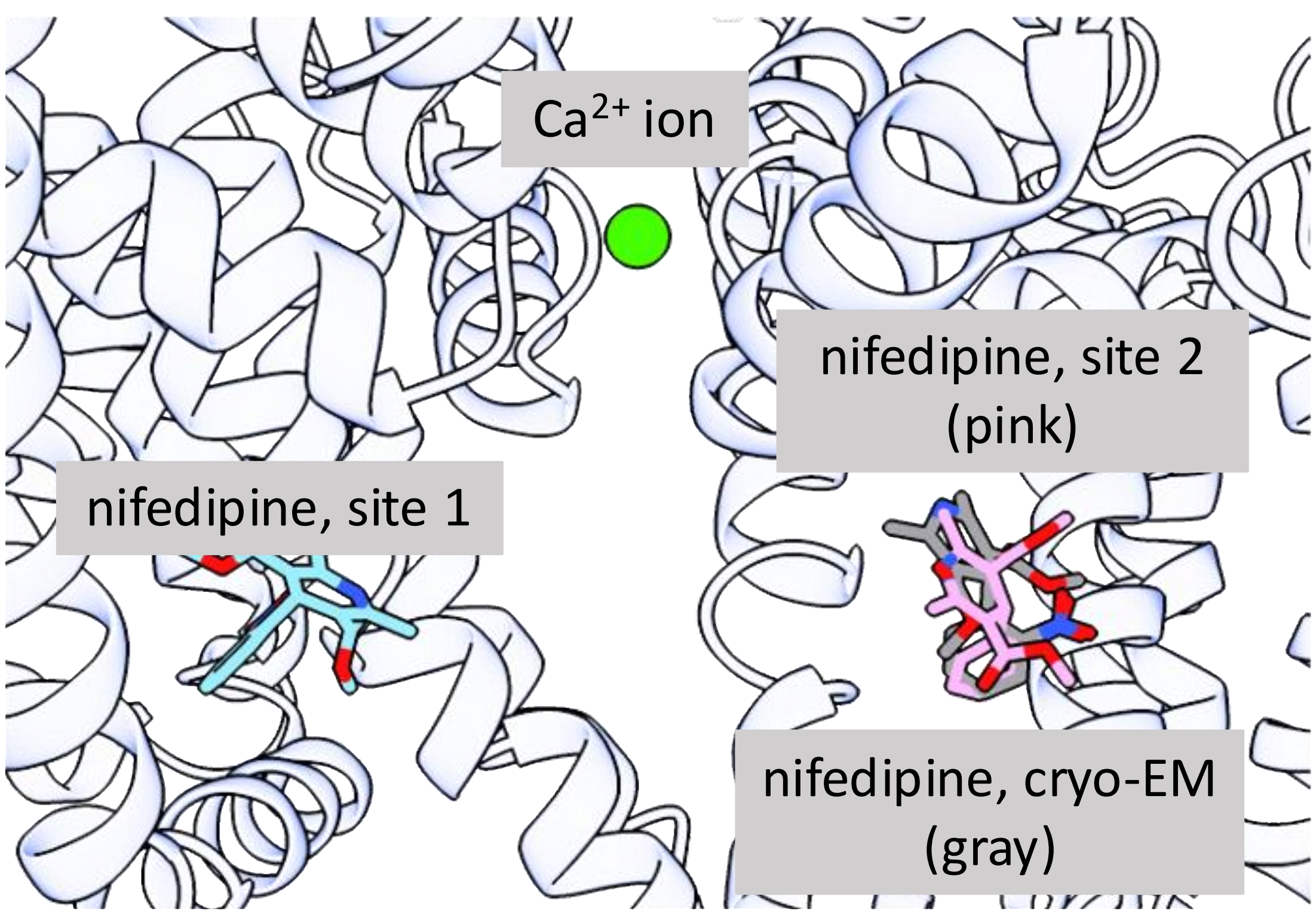

	docking score (kcal/mol)	
**drug**	**Site 1**	**Site 2**
nifedipine	-9.901	-8.321
nitrendipine	-10.119	-10.262

Figure S8: **Ca_V1.2 channel drug docking results using OpenEye FRED.** (A) OpenEye docking scores for the 53 drugs from Kramer et al. (main text ref. 19) to Site 1 of the Ca_V1.2 channel (PDB: 8FHS). Site 1 is the result of broad docking within the entire intracellular cavity, which results in a minimum-energy pose near the fenestration between domains I and II. (B) OpenEye docking of the same drug set to Site 2 of the same Ca_V1.2 channel model is the result of a narrower search area, focused on the dihydropyridine (DHP) binding site, the fenestration between domains III and IV. (C) Docking score correlation taking the lower (more favorable) OpenEye docking score values from site 1 and 2 vs. experimental IC_{50} values. Scores for nifedipine and nitrendipine are circled in each panel. (D) Nifedipine docking poses produced by OpenEye FRED in two different fenestration sites. Cryo-EM pose of nifedipine is from the rabbit Ca_V1.1 channel structure (PDB: 6JP5). The docking score for nitrendipine, a related DHP antagonist, also does not differ significantly between the two sites.

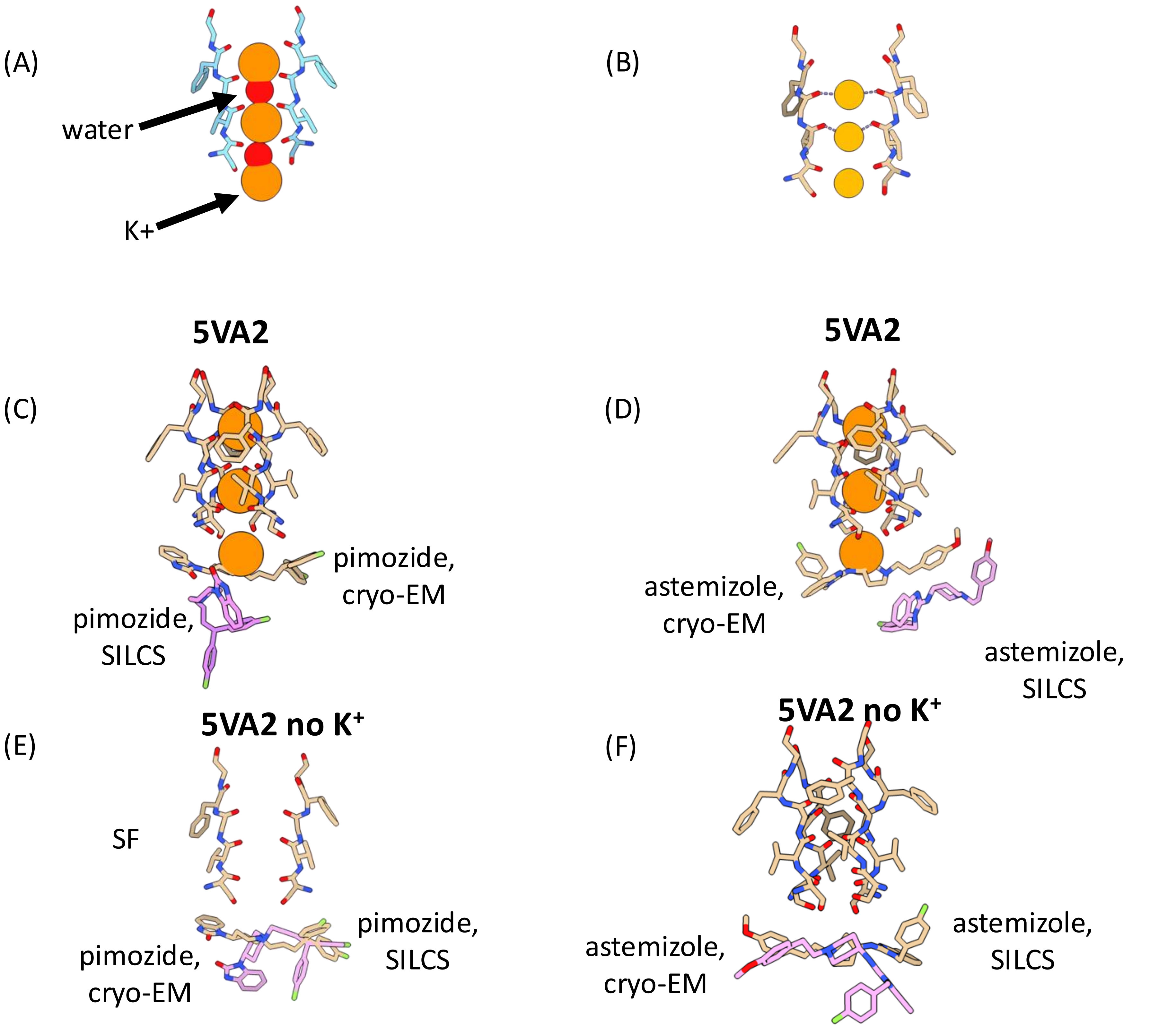

***Figure S9*: hERG channel - drug interactions.** (A) K^+ ions and SF waters used with hERG PDB: 5VA2 models. (B) SF K^+ ions from PDB: 7CN1. (C-F) SILCS docked (pink) and experimental (brown) orientations of (C) pimozide: RMSD (non hydrogens) = 8.8 Å; (D) astemizole: RMSD = 10.6 Å; (E) pimozide: RMSD = 4.6 Å. (F) astemizole: RMSD = 8.3 Å. (C) and (D) show docking poses with K^+ ions while (E) and (F) show poses without K^+ ions, which leads to lower RMSDs with respect to cryo-EM binding poses (PDB: 8ZYO for astemizole and PDB: 8ZYQ for pimozide).

Model terms	ROC AUC	Sensitivity	Specificity	Accuracy
hERG IC50	0.852	0.774	0.864	0.811
hERG LGFE	0.811	0.970	0.591	0.811
hERG + Cav IC50	0.871	0.818	0.880	0.849
hERG + Cav LGFE	0.886	0.645	0.955	0.774
hERG + Nav + Cav IC50	0.870	0.871	0.773	0.830
hERG + Nav + Cav LGFE	0.943	0.903	0.909	0.906

Figure S10: **Logistic regression analysis model performance using leave-one-out cross validation on 53-drug training set.** IC_{50} indicates experimental values from Kramer et al. (main-text ref. 19) and LGFE indicates SILCS docking score to the respective ion channel models: hERG_5VA2, Cav_6AGF, and Nav_6LQA.

ML algorithm	Hyperparameters	Value range tested	Final value
Random Forest (RF)	n_estimators	(50, 300)	74
	max_depth	(3, 30)	24
	min_samples_split	(2, 20)	3
	min_samples_leaf	(1, 20)	1
XGBoost (XGB)	n_estimators	(50, 300)	193
	max_depth	(3, 10)	9
	learning_rate	(0.01, 0.2)	0.09
	subsample	(0.6, 1)	0.68
	colsample_bytree	(0.6, 1)	0.77
	reg_alpha	(0.0, 1)	0.19
	reg_lambda	(1, 3)	1.05
Neural Network (NN)	hidden1	(32, 128)	124
	hidden2	(16, 64)	64
	dropout	(0.1, 0.5)	0.448
	learning_rate	(0.0001, 0.01)	0.0073
	weight_decay	(0.000001, 0.01)	0.00085
	n_epochs	100	100

Figure S11: **Hyperparameters from machine learning (ML) classifier models.** Values were varied uniformly within the range listed by the Optuna software package to optimize the area under the receiver operating characteristic Curve (AUC ROC) of each model.

(A) $hERG_{5VA2} + \boxed{Cav_{6AGF}} + hERG_{S641A}$ vs $hERG_{5VA2} + \boxed{logP,} + hERG_{S641A}$

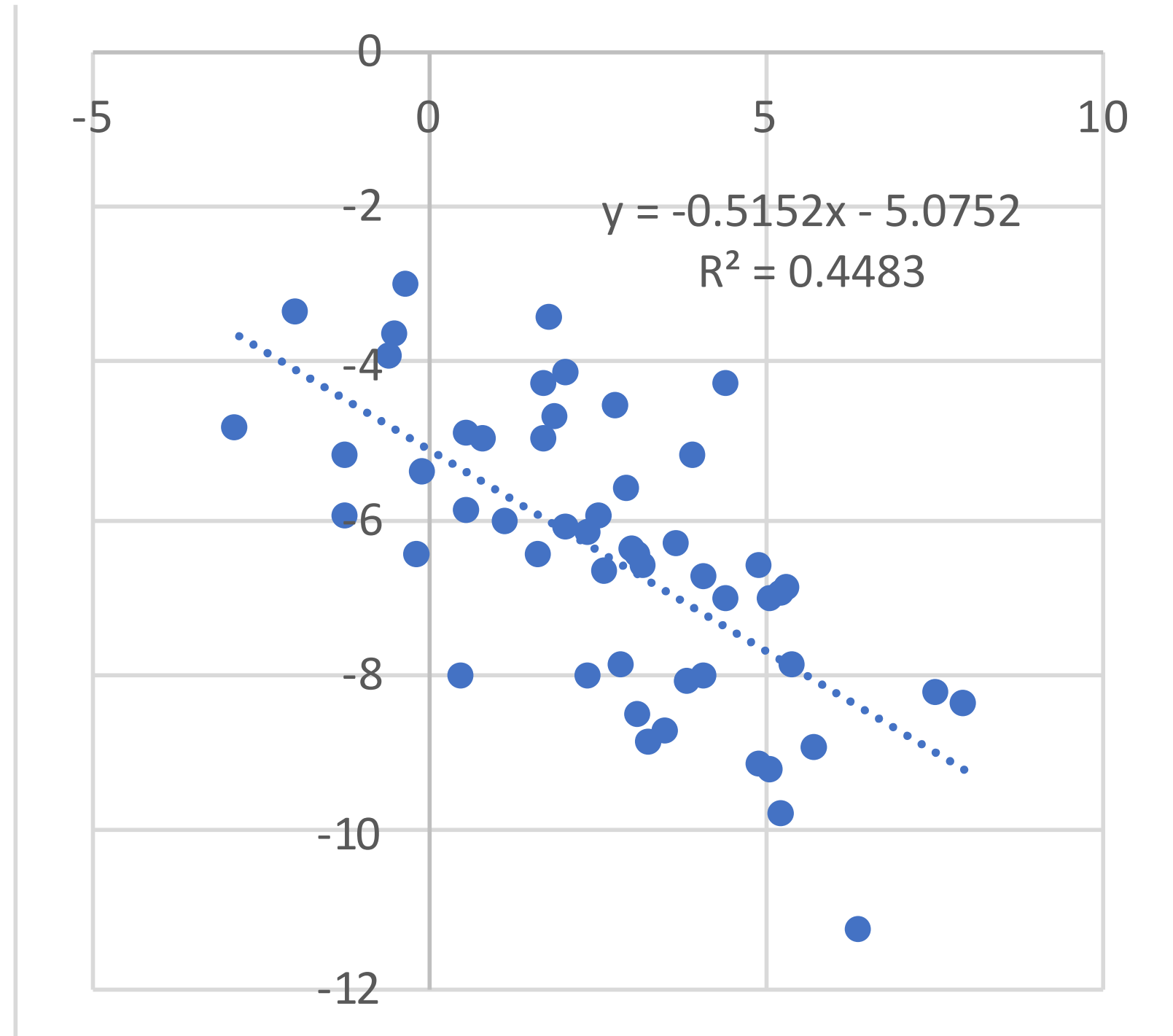

(B)

$hERG_{open} + Cav_{6AGF} + \boxed{hERG_{9CHQ}}$ vs $hERG_{5VA2} + Cav_{6AGF} + \boxed{Nav_{6LQA}}$

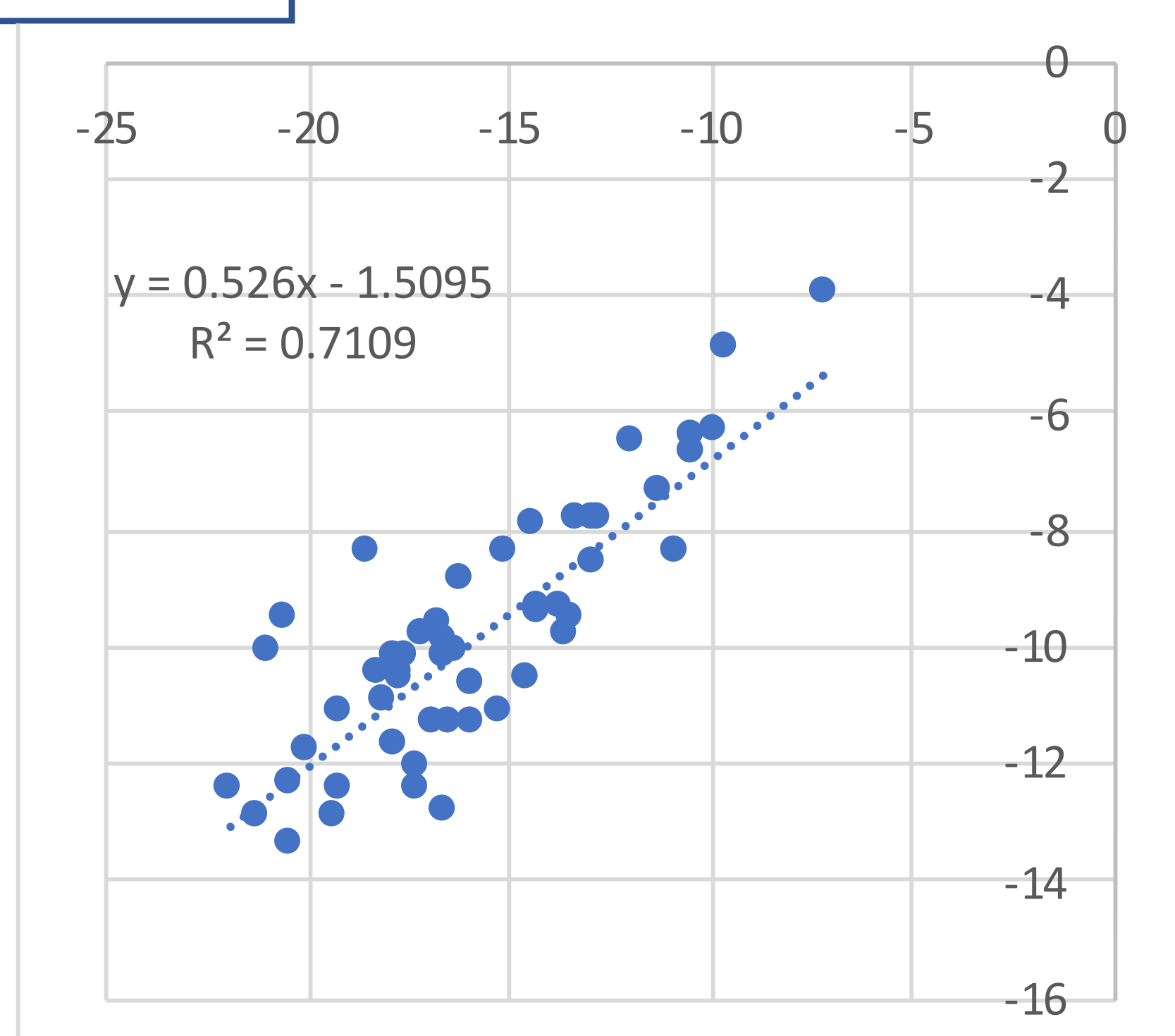

Figure S12: **Correlation between different TdP risk prediction model scores.** (A) Correlation between open-state Ca_V1.2 LGFE scores (Cav_{6AGF}) and log *P* values and (B) correlation between between low K^+ hERG LGFE scores ($hERG_{9CHQ}$) and inactivated Na_V1.5 LGFE score (Nav_{6LQA}).

Topological Index	Description
Balaban J Index (J)	$$J = \frac{N}{M - N + 2} \sum_{bonds\ i<j} \left(s_i s_j\right)^{-1/2}$$ N=number of heavy atoms, M=number of bonds. s_i=sum of the distances from atom i to every other atom (measured in number of bonds). High values of J indicate linear molecules while low values indicate more branched or cyclized molecules.
Kappa1 (κ_1)	$$\kappa_1 = \frac{(N-1)^2}{M^2}$$ High values indicate long, linear molecules with minimal branching.
Kappa2 (κ_2)	$$\kappa_2 = \frac{(N-1)(N-2)^2}{P_2^2}$$ P_2=number of paths with length two. High values indicate a molecule with a high degree of branching.
Kappa3 (κ_3)	$$\kappa_3 = \frac{(N-1)(N-3)^2}{P_3^2}$$ P_3=number of paths with length three. High values indicate a molecule with a high number of cycles/rings.
Bertz Complexity Index (C_T)	$$C_T = \ln\left[\prod_{i=1}^{N}(d_i)!\right]$$ d_i=number of atoms directly bonded to atom i. High values of C_T indicate more complex molecules with a high degree of branching and/or cycles.

Figure S13: **Description of topological indices used as machine-learning classifier model features.**

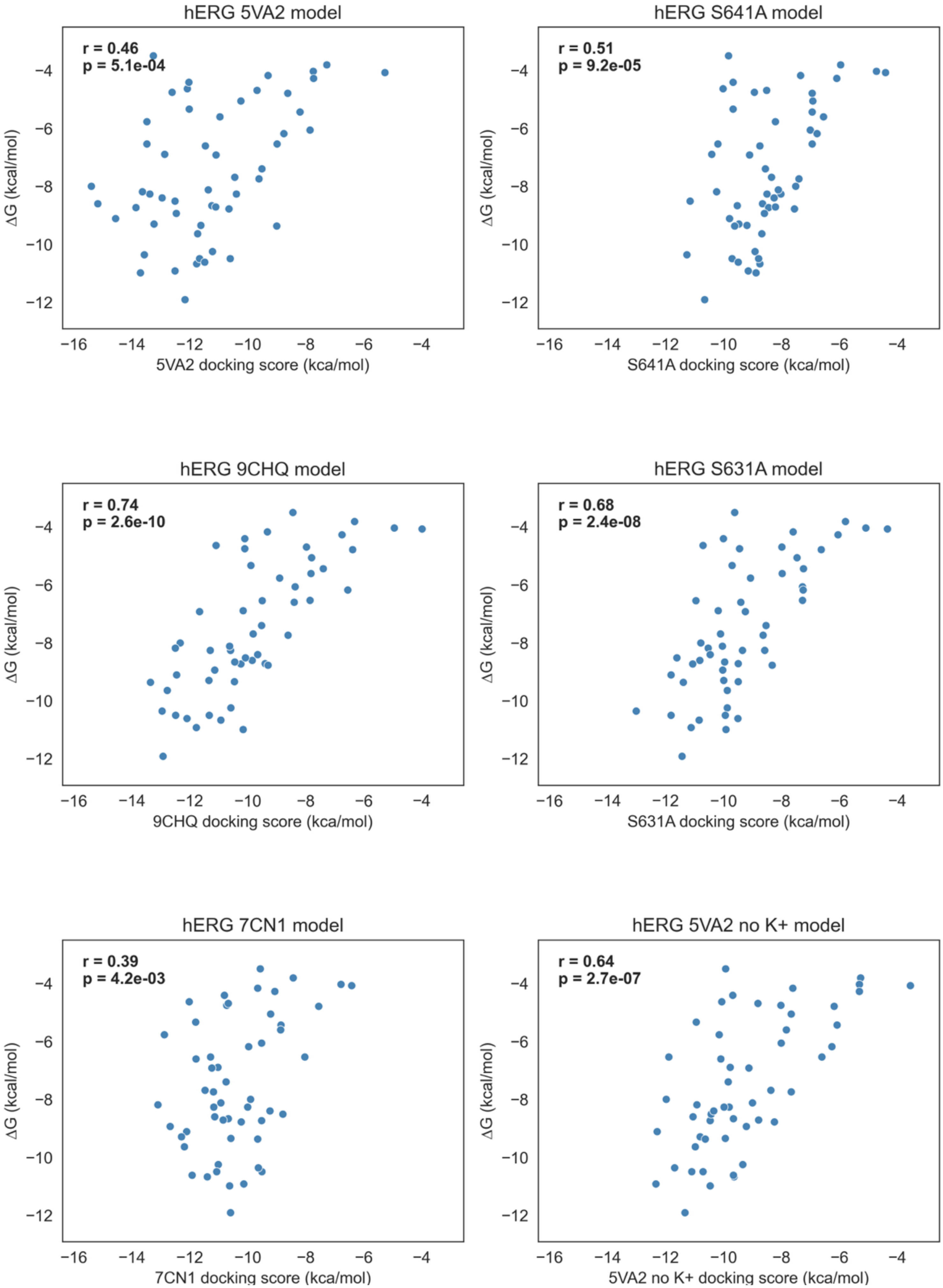

Figure S14: **Correlation of SILCS LGFE scores and experimental data using different hERG channel models**. Experimental Δ*G* values from the reported IC_{50} values are shown on the *Y* axes and SILCS LGFE docking scores are shown on the *X* axes. The *p*-values were computed using two-tailed Pearson correlation as in Figure S7.

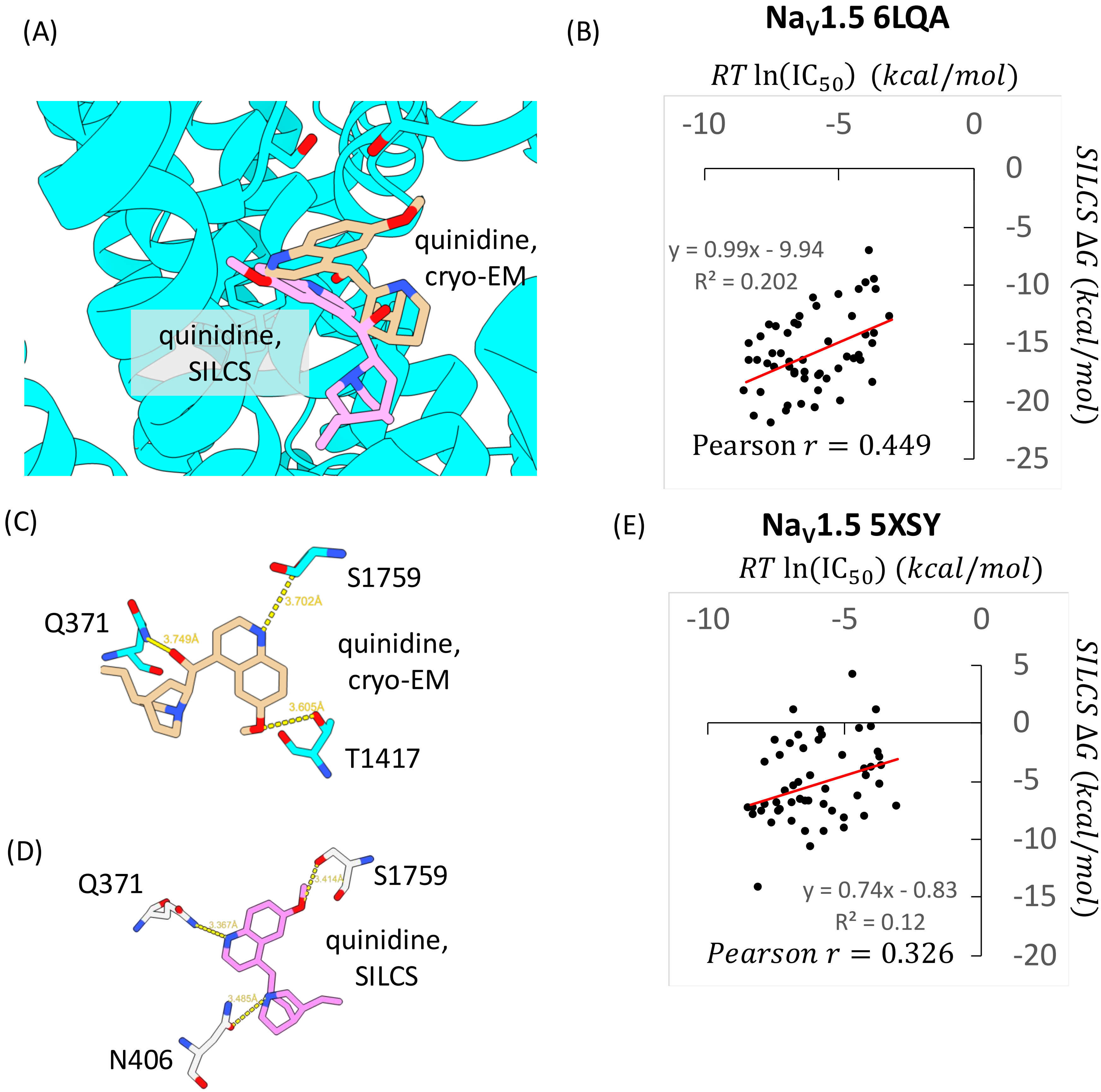

Figure S15: **Na_V1.5 channel-drug interactions.** (A) SILCS quinidine binding pose (pink) compared to cryo-EM quinidine (QDN) pose (brown) from PDB: 6LQA, RMSD (non-hydrogen) = 5.22 Å. (B) Correlation between experimental IC_{50} values from Kramer et al. (main-text ref. 19) and SILCS LGFE scores for 53 drug test set for PDB: 6LQA. IC_{50} values were converted to kcal/mol as $RT\ \ln(IC_{50})$. (C-D) QDN contacts from SILCS binding pose include the tertiary amine contact with Na_V1.5 residue N406 instead of T1417 with QDN hydroxyl from cryo-EM PDB: 6LQA. (E) Correlation between experimental IC_{50}-s from Kramer et al. (main-text ref. 19) and SILCS docking scores for 53 drug test set for Na_V1.5 homology model based on PDB: 5XSY.

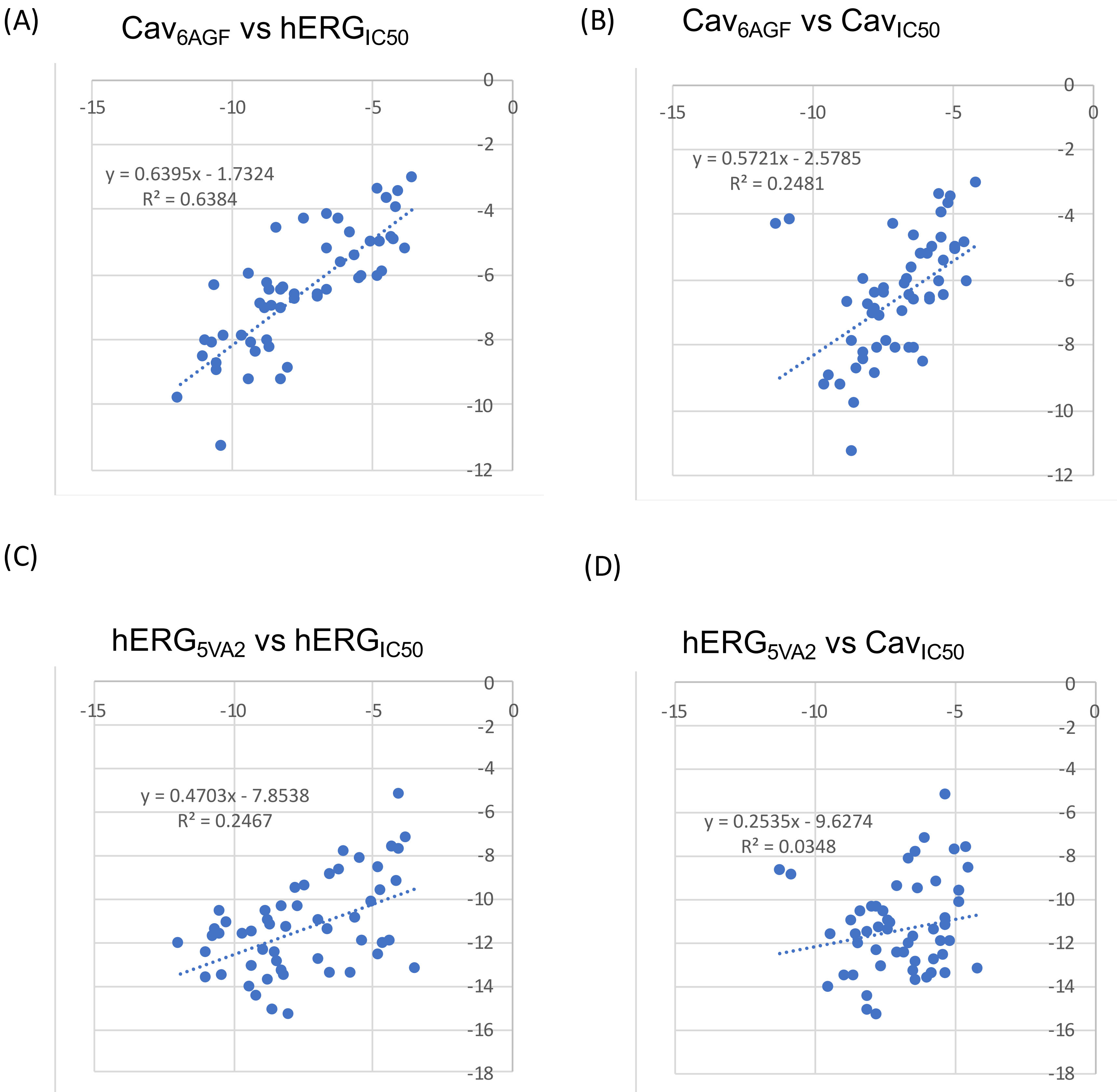

***Figure S16:* Correlation between SILCS LGFE scores and experimental Δ*G* values derived from IC_{50}-s.** (A) Open-state $Ca_V1.2$ (Cav_{6AGF}) LGFEs vs experimental hERG Δ*G*-s ($hERG_{IC50}$) (B) Cav_{6AGF} vs experimental $Ca_V1.2$ Δ*G*-s (Cav_{IC50}) (C) Open-state hERG LGFE scores ($hERG_{5VA2}$) vs $hERG_{IC50}$ and (D) $hERG_{5VA2}$ vs Cav_{IC50}. All Δ*G* values are in kcal/mol.

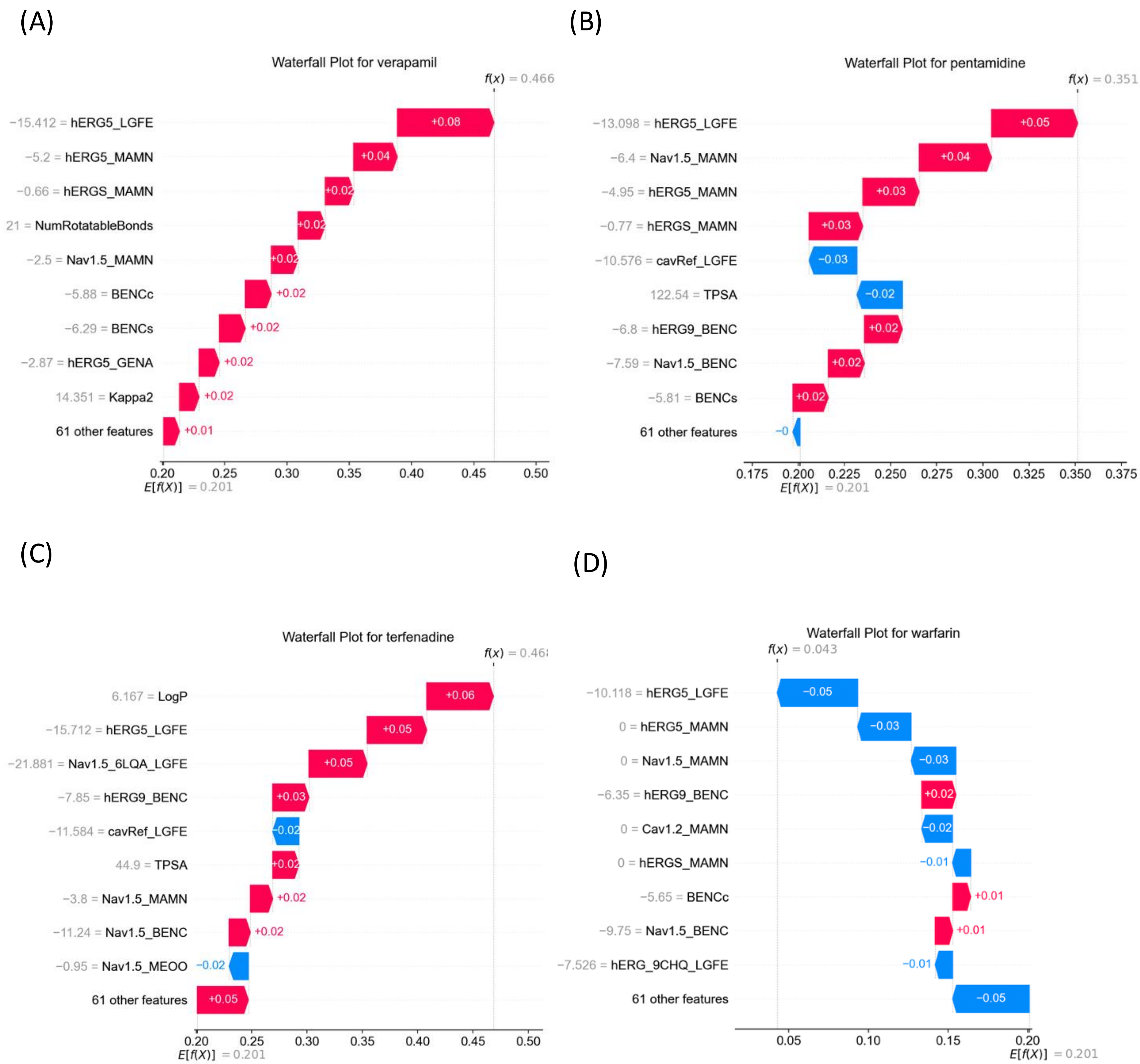

Figure S17: **Waterfall plots showing feature importance from random forest classifier model** for (A) verapamil (B) pentamidine (C) terfenadine, and (D) warfarin.
